# Oral administration of *Pinus koraiensis* cone essential oil reduces rumen methane emission by altering the rumen microbial composition and functions in Korean native goat (*Capra hircus coreanae*)

**DOI:** 10.3389/fvets.2023.1168237

**Published:** 2023-05-18

**Authors:** Youyoung Choi, Shin Ja Lee, Hyun Sang Kim, Jun Sik Eom, Seong Uk Jo, Le Luo Guan, Jakyeom Seo, Tansol Park, Yookyung Lee, Sang Suk Lee, Sung Sill Lee

**Affiliations:** ^1^Division of Applied Life Science (BK21), Gyeongsang National University, Jinju, Republic of Korea; ^2^Institute of Agriculture and Life Science (IALS), Gyeongsang National University, Jinju, Republic of Korea; ^3^Institute of Agriculture and Life Science and University-Centered Labs, Gyeongsang National University, Jinju, Republic of Korea; ^4^Department of Agricultural, Food and Nutritional Science, University of Alberta, Edmonton, AB, Canada; ^5^Department of Animal Science, Life and Industry Convergence Research Institute, Pusan National University, Miryang, Republic of Korea; ^6^Department of Animal Science and Technology, Chung-Ang University, Anseong, Republic of Korea; ^7^Animal Nutrition and Physiology Team, National Institute of Animal Science, RDA, Jeonju, Republic of Korea; ^8^Ruminant Nutrition and Anaerobe Laboratory, Department of Animal Science and Technology, Sunchon National University, Sunchon, Republic of Korea

**Keywords:** enteric methane emission, essential oil, feed additives, metataxonomic, rumen microbiota

## Abstract

This study aimed to investigate *Pinus koraiensis* cone essential oil (PEO) as a methane (CH_4_) inhibitor and determine its impact on the taxonomic and functional characteristics of the rumen microbiota in goats. A total of 10 growing Korean native goats (*Capra hircus coreanae*, 29.9 ± 1.58 kg, male) were assigned to different dietary treatments: control (CON; basal diet without additive) and PEO (basal diet +1 g/d of PEO) by a 2 × 2 crossover design. Methane measurements were conducted every 4 consecutive days for 17–20 days using a laser CH_4_ detector. Samples of rumen fluid and feces were collected during each experimental period to evaluate the biological effects and dry matter (DM) digestibility after PEO oral administration. The rumen microbiota was analyzed via 16S rRNA gene amplicon sequencing. The PEO oral administration resulted in reduced CH_4_ emission (eructation CH_4_/body weight^0.75^, *p* = 0.079) without affecting DM intake; however, it lowered the total volatile fatty acids (*p* = 0.041), molar proportion of propionate (*p* = 0.075), and ammonia nitrogen (*p* = 0.087) in the rumen. Blood metabolites (i.e., albumin, alanine transaminase/serum glutamic pyruvate transaminase, creatinine, and triglyceride) were significantly affected (*p* < 0.05) by PEO oral administration. The absolute fungal abundance (*p* = 0.009) was reduced by PEO oral administration, whereas ciliate protozoa, total bacteria, and methanogen abundance were not affected. The composition of rumen prokaryotic microbiota was altered by PEO oral administration with lower evenness (*p* = 0.054) observed for the PEO group than the CON group. Moreover, PICRUSt2 analysis revealed that the metabolic pathways of prokaryotic bacteria, such as pyruvate metabolism, were enriched in the PEO group. We also identified the Rikenellaceae RC9 gut group as the taxa potentially contributing to the enriched KEGG modules for histidine biosynthesis and pyruvate oxidation in the rumen of the PEO group using the FishTaco analysis. The entire co-occurrence networks showed that more nodes and edges were detected in the PEO group. Overall, these findings provide an understanding of how PEO oral administration affects CH_4_ emission and rumen prokaryotic microbiota composition and function. This study may help develop potential manipulation strategies to find new essential oils to mitigate enteric CH_4_ emissions from ruminants.

## Background

Climate warming caused by methane (CH_4_) is one of the most pressing environmental issues (1). Methane has a 28-fold greater global warming potential compared to carbon dioxide. In 2020, global anthropogenic CH_4_ emissions were 380 Mt. of which 49 Mt./year was emitted from livestock (2). Enteric CH_4_ emissions in ruminants contribute to more than 95% of total livestock CH_4_ emissions (3), and most CH_4_ from ruminants is generated by rumen microbial methanogenesis (4). In addition, the energy lost as CH_4_ from ruminants ranges between 2% and 12% of gross energy intake (5). Therefore, reducing CH_4_ emissions has direct economic benefits because it coincides with greater energy-use efficiency of animal feed (6).

Many nutritional manipulation strategies have been implemented to persistently reduce CH_4_ emissions in ruminants, improve animal feed efficiency, and minimize dietary energy loss. Essential oils (EOs) are natural compounds that can be used in livestock feed (7). EOs are volatile compounds produced by plants and contain various chemical substances (7, 8). EOs have shown a wide range of antimicrobial properties against rumen microbiota, including ciliate protozoa and fungi; however, there is also evidence of their benefits on rumen fermentation (9–11). For example, EO can reduce CH_4_ emissions by suppressing the abundance and diversity of methanogens and can reduce ammonia nitrogen excretion (12–15). In addition, EO can stimulate the immune system, thereby enhancing the resistance of animals to inflammatory and infectious diseases. Yesilbag et al. (16) have shown that supplementation with juniper oil (0.4–2 mL/kg of DM, containing 89.7% α-pinene) significantly increased the activities of superoxide dismutase, catalase, and total antioxidant in growing goats. Moreover, garlic oil (2 g/d) into the abomasum increased T helper cells related to adaptive immunity without affecting the intestinal microbial abundance in dairy cows (17). However, although the effect of EOs on ruminants is well reported, the effect of EOs on the composition and function of the rumen microbiome remains unclear.

In a previous study, the beneficial effects of *Pinus koraiensis* cone essential oil (PEO) were confirmed using a series of *in vitro* evaluation systems (18). Specifically, PEO was found to contain two primary active compounds, α-pinene and D-limonene, which accounted for 38.6% and 30.7% of the total proportion of compounds, respectively. Furthermore, previous research has demonstrated that both compounds can reduce rumen methane production (19). The study also suggested that the reduction in CH_4_ emission caused by PEO supplementation is likely due to changes in rumen fermentation rather than selective alterations in the abundance of rumen methanogens. Thus, in this study, we hypothesized that PEO oral administration might affect rumen metabolism. The aim was to investigate how PEO affects rumen microbial composition and function and its potential to mitigate rumen CH_4_ emissions by altering rumen fermentation in goats. This study might provide information that could be used to develop effective CH_4_ mitigation strategies and help improve our understanding of how PEO oral administration affects the rumen microbiota community and function.

## Materials and methods

### Ethics statement

All experimental procedures were reviewed and approved by the Gyeongsang National University Institutional Animal Care and Use Committee (protocol number: GNU-210705-E0063). The experiment was carried out at the Gyeongsang National University Animal Breeding Farm from 10 January to 08 March 2022.

### Sample information

The PEO extracted from *Pinus koraiensis* cones was provided by PHYLUS (PHYLUS Co., Ltd. Seoul, Korea). In brief, pinecones were cut in a range of 1–4 cm size, and 100 g of pinecones was moved into a 1 L round-bottomed flask. Afterward, pinecones were mixed with 500 mL of distilled water for 2 min, and then a steam distillation process (100°C, 1 h) was performed. After cooling the extract (16°C–20°C), the essential oil layer was finally separated and purified by microfiltration. The detailed PEO extraction method was the same as that used by Kim and Lee (20). The chemical composition of the EO was previously analyzed by Choi et al. (18) using gas chromatography–mass spectrometry (GC-2010 PLUS, GC–MS-TQ 8030, Shimadzu, Tokyo, Japan) equipped with DB-5MS column (I.D. 0.25 mm, L.30 m) which was used to obtain a total of 43 individual compounds. The detailed composition of PEO is described in our previous study (18).

### Experimental design, animals, and diet

A total of 10 growing Korean native goats (*Capra hircus coreanae*, 29.9 ± 1.58 kg, male) were kept in individual 170 × 120 cm pens for experiments. Goats were randomly assigned to dietary treatments which were (1) control (CON; basal diet without additive) and (2) *Pinus koraiensis* cone essential oil (PEO, basal diet +1 g/d of PEO) by 2 × 2 crossover design (two groups at two different time periods). The oral administration dosages of PEO were determined based on a previous *in vitro* assay (18). To ensure that the full dose of PEO was received, the liquid form of the PEO was orally delivered in 5 mL of water to individual goats using a 10-mL syringe, before their morning feeding. To enable adjustments among the treatments, the CON group received an oral administration of 5 mL of water. Animals were fed the same diet composed of tall fescue and a commercial concentrate. The chemical composition of the tall fescue and commercial concentrate are presented in Table 1. Diet and PEO additives were provided to animals in two equal meals at 08:00 and 16:00 h. The experimental diet consisted of tall fescue hay and concentrate provided at a ratio of 50:50 to meet nutrient requirements according to NRC (21) recommendations. Tall fescue hay was given before the concentrate mix at each feeding to make the goats consume forage as much as possible before ingesting concentrates. Drinking water was offered *ad libitum*. Individual daily feed intake was recorded by measuring feed offered and refusals. Each of the two experimental periods lasted for 21 days (i.e., 16 days of adaptation and 5 days of data and sample collection) and 14 days wash-out between periods.

**Table 1:** Mineral and chemical composition of experimental diets fed to goats.

Item^1^	Tall fescue	Concentrate^2^
**Mineral composition (% of DM)**		
Ash	10.9	9.46
Calcium	0.50	1.25
Phosphorus	0.20	0.66
Magnesium	0.21	0.32
Potassium	2.27	1.24
Sodium	0.12	0.36
Iron, mg/kg	186	366
Manganese, mg/kg	47.0	128
Zinc, mg/kg	14.0	174
Copper, mg/kg	5.0	34.0
**Chemical composition (% of DM)**		
Dry matter	91.7	89.5
Moisture	8.30	10.5
Crude protein	7.50	16.4
Soluble protein	2.31	6.22
Ether extract	1.52	4.14
Starch	1.50	-
NDF	56.7	33.7
NDICP	1.23	4.37
ADF	35.3	16.6
ADICP	0.72	1.25
Lignin	3.73	4.50
Non-fiber carbohydrates^3^	23.4	36.3
**Energy, Mcal/lb**		
Metabolizable energy	0.98	1.17
Net energy maintenance^4^	0.59	0.76
Net energy gain	0.33	0.49
Total digestible nutrients (% of DM)	58.6	68.2

SEM, standard error of the mean; DM, dry matter. NDF, neutral detergent fiber; NDICP, neutral detergent insoluble crude protein; ADF, acid detergent fiber; ADICP, acid detergent insoluble crude protein.

1 Chemical analysis was performed by Cumberland Valley Analytical Services (Waynesboro, PA, United States).

2 The formulation of the concentrate used in this study contained crude protein (13.5% or more), ether extract (2.5% or more), crude fiber (20.0% or less), ash (10.0% or less), calcium (0.80% or more), phosphate (0.80% or less), and TDN 67% (values are concentrations declared by the manufacturer).

3 According to Hall’s (22) equation.

4 Net energy for maintenance (NEm) concentration was calculated using the OARDC Summative Energy Equation of Weiss (23).

### Methane measurements and data processing

Enteric CH_4_ emissions were measured using a laser methane detector (LMD; LMm-G; Tokyo Gas Engineering Co. Ltd., Tokyo, Japan) as described by Roessler et al. (24) and Kang et al. (25) with minor modifications. In brief, every 4 consecutive days (17–20 days), CH_4_ emissions were measured twice a day: before feed intake: 06:00–08:00 h and after feed intake: 09:00–11:00 h. Methane concentration in the breathing air was recorded from ppm-m at an interval of 0.5 s for 8 min. The operator continuously adjusted the LMD to point to the visible laser on the same spot following the animal’s head movement. For all measurements, a fixed distance of 1 m was allowed between the goat and LMD. Eructation, the primary track of CH_4_ emissions, coincides with the B-sequence of rumen contractions, which occur irregularly once every 1–3 min (26). Therefore, the measurement duration was set to 8 min, which was long enough to capture 3–4 eructation, as suggested by Kang et al. (25). This was similar to the suggested duration (minimum 2 min) for keeping an animal in the GreenFeed system to ensure the inclusion of at least one eructation event (27).

The measured CH_4_ concentrations were calculated according to the method of Kang et al. (25). In brief, the peaks of CH_4_ concentration measured by LMD were detected using the *AMPD* package in R. Detected peaks were divided into two tracks (respiration and eructation) by fitting a double normal distribution using the *mixdist* R package. A larger number of peaks belonged to respiration; however, their value was low. The mean of the normal distribution was assumed to be the representative CH_4_ concentration of the gas exhaled from the track for the hour. The values of CH_4_ concentration measured four times a day were averaged to represent the mean daily CH_4_ concentration.

### Feed sampling and analyses

Dried feed samples (tall fescue and concentrate) were ground through a 1-mm sieve using a Wiley Mill (Arthur Thomas CO., Philadelphia, PA) and were analyzed for dry matter (DM, AOAC International, 2000 (28); method 930.15), crude protein (CP, AOAC International, 2000 (28); method 990.03), ether extract (EE, AOAC International, 2006 (29); method 2003.05), ash (AOAC International, 2000 (28); method 942.05), minerals (AOAC International, 2000 (28); method 985.01), amylase-treated neutral detergent fiber (aNDF) (30), acid detergent fiber (ADF, AOAC International, 2000 (28); method 973.18), neutral detergent insoluble crude protein (NDICP, LecoFP-528 N Combustion Analyzer), acid detergent insoluble crude protein (ADICP, LecoFP-528 N Combustion Analyzer), lignin (30), and starch (22) by Cumberland Valley Analytical Services (Waynesboro, PA). Non-fiber carbohydrates (NFCs) were calculated according to the equation by Hall (31); NFC = 100 – [(CP – NDICP) + ether extract + ash + NDF]. Net energy for maintenance (NEm) was calculated using the OARDC Summative Energy Equation, as described by Weiss (23).

### Ruminal sampling and analyses

Rumen contents were collected with oral stomach tubing (length of 150 cm and a diameter of 0.8 cm) from each animal before morning feeding and once at the end of each experimental period. In brief, samples of rumen contents were collected by inserting an oral stomach tube to a depth of approximately 20 cm, so that the probe head could reach the central rumen. To minimize contamination from the saliva, the first 20 mL of each rumen fluid sample was discarded. The rumen fluid from each goat was collected and filtered using a cheesecloth folded into four layers. Subsequently, 5 mL of the filtered rumen fluid was transferred into a 15-mL Falcon tube, and the pH was measured using a pH meter (S220, Mettler-Toledo, Greifensee, Switzerland). Filtered rumen fluid (10 mL) was divided into two separate aliquots: analysis for volatile fatty acid (VFA) and ammonia nitrogen (NH_3_-N). Filtered rumen fluid (5 mL) was centrifuged at 20,000 × *g* for 15 min at 4°C, the supernatant was discarded, and the pellet was stored at −80°C for microbial analysis. All the aliquots were transported to the laboratory (with dry ice) and stored under −80°C until subsequent analysis.

For the VFA processing, 1 mL of rumen fluid samples were centrifuged at 20,000 × *g* for 15 min at 4°C, and the supernatant was used to analyze with a high-performance liquid chromatography (L-2200, Hitachi, Tokyo, Japan) equipped with a UV detector (L-2400; Hitachi) and a column (MetaCarb 87H; Varian, Palo Alto, CA, United States) according to Adesogan et al. (32). The NH_3_-N concentration was measured by optical density at 630 nm by spectrophotometer (Model 680, Bio-Rad Laboratories, Hercules, CA, United States) following the protocol described by Chaney and Marbach (33).

### Blood sampling and analysis

On day 20 of each sampling period before the morning feeding, blood from the jugular neck vein was collected in a serum-separating tube (BD Vacutainer, SST™ II advance, Becton Dickinson Co., Franklin Lakes, NJ, United States) from the goats. The blood samples were centrifuged for 15 min at 1,006 × *g* at 4°C, and the serum was stored at −80°C until analysis. The contents of serum alanine transaminase/serum glutamic pyruvate transaminase (ALT/SGPT), aspartate aminotransferase/serum glutamic oxaloacetic transaminase (AST/SGOT), inorganic phosphate, and glucose were measured using the UV spectrophotometry method by a Cobas 8000 c702 analyzer (Roche Diagnostics, Mannheim, Germany). The contents of albumin, total cholesterol, creatinine, total protein, triglyceride, and calcium were measured using the colorimetric method by a Cobas 8000 c702 analyzer (Roche Diagnostics, Mannheim, Germany).

### Fecal sampling and analysis

Total fecal samples were collected to estimate DM digestibility percentage. In brief, total fecal collection was performed three times during each experimental period on a 12-h basis. Within 6 h of defecation, each fecal sample was weighed and placed in a bag for each goat during each period. To prevent urine from mixing with the fecal samples, three diapers (Guards for seniors, Depends XL size, Yuhan-Kimberly Inc., Seoul, Korea) were attached to each goat using an extra-large washable male canine wrap (34). When the diapers were wet, they were replaced with new diapers. Fecal samples were dried in a forced-air oven for 96 h at 55°C until the weight no longer changed.

### DNA extraction and real-time PCR analysis

The total DNA of rumen fluid (1.8 mL) was extracted using the RBB + C method (35). The quality and quantity of extracted DNA were evaluated using a NanoDrop ND-2000 spectrophotometer (Thermo Fisher Scientific Inc., Waltham, MA, United States). Extracted DNA was used for real-time PCR (CFX 96 Touch system, Bio-Rad Laboratories, Inc.) to quantify the abundance of rumen representatives including total bacteria, ciliate protozoa, fungi, and methanogens. The primers used for real-time PCR are presented in Supplementary Table S1. Primer sets and real-time PCR conditions used were the same as reported for general bacteria (36), fungi (36), protozoa (37), and methanogens (38). In brief, plasmid DNA containing the respective target bacterial 16S rRNA gene sequence was obtained by PCR cloning using the bacteria-specific primer set. Copy number of each standard plasmid was calculated using the molecular weight of nucleic acid and the length of the cloned standard plasmid. In total, 10-fold dilution series ranging from 1 to 10^10^ copies were prepared for each target and run along with the samples. To quantify each gene, we used a standard curve generated by amplifying a dilution series of plasmid DNA that contained the corresponding target sequence. All the detailed procedures of PCR condition and manufacturing of each microbe plasmid were proceeded according to Kim et al. (39) and Choi et al. (40).

### Metataxonomic analysis of the rumen prokaryotic microbiota

Amplicons targeting the V3–V4 region of 16S rRNA genes were prepared and sequenced (Macrogen, Seoul, Korea). In brief, indexed 16S rRNA gene amplicon libraries from both bacteria and archaea were amplified using 341F (5′-CCTACGGGNGGCWGCAG-3′) and 805R (5′-GACTACHVGGGTATCTAATCC-3′) universal primers (41) with a unique barcode for each DNA sample. The obtained amplicon was sequenced using the Illumina MiSeq platform (San Diego, CA, United States), and QIIME2 (version 2021.11) (42) was used to analyze sequence data. After demultiplexing the sequences, the barcode and primer sequences were removed using Cutadapt (43). Afterward, the DADA2 plugin was used to denoise the forward and reverse reads with quality filtering (Q-score > 25) and merged, which was followed by chimera removal (44). Subsequently, de-noised sequences were clustered to the amplicon sequence variants (ASVs) using the naïve Bayes taxonomy classifier manually trained on Silva (SSU138) 16S rRNA gene database (clustered at 99% similarity; 341F/805R region) (45). ASVs were excluded when identified as unassigned mitochondria and chloroplast before downstream analysis. To reduce the sampling heterogeneity, the ASV table was rarefied to the same reads per sample (39,057 ASVs) 1,000 times using the “q2-repeat-rarefy” plugin from QIIME2 (46). Microbial diversity was evaluated within samples (alpha diversity) or between samples (beta diversity) on the rarefied ASV table. Alpha diversity was evaluated using richness (Chao1 estimates), Evenness, Simpson’s index, and Shannon’s index. Beta diversity was evaluated using the phylogenetic distance of Bray–Curtis and Weighted UniFrac. Prediction of metabolic functions (KEGG modules and pathways) from the rumen prokaryotic microbiota was performed using the PICRUSt2 tool (v.2.4.1) (47).

To understand the relationship among the taxonomic groups of the major prokaryotic genera (relative abundance ≥ 0.01%) in the CON and PEO groups, the co-occurrence network analysis was generated using the “FastSpar” (48) which uses the SparCC algorithm (49). Functional Shifts’ Taxonomic Contributors software (FishTaco v1.1.6) (50) was used to identify the rumen prokaryotic microbiota driving the functional shifts between the CON and PEO groups. The genus level of taxonomic abundance and functional abundance profiles from the KEGG module was used as input files for the FishTaco analysis. The visualization of the output was performed in the FishTaco Plot package in R. The raw amplicon sequences from this study were deposited in the NCBI Sequence Read Archive (accession numbers: PRJNA887912).

### Statistical analysis

Statistical analysis was performed using SAS (version 9.4, SAS Institute Inc., Cary, NC) and R software (version 4.0.2). Initially, the mathematical assumptions data were tested using the Shapiro–Wilk test. The data obtained from *in vivo* experiment were analyzed using the PROC GLIMMIX procedure according to the following statistical model:


$$Y_{ijk}=\mu +A_{i}+P_{j+}+T_{k}+{\left(PT\right)}_{jk}+\varepsilon_{ijk}$$

where Y_ijk_ = observed dependent variable, μ = overall mean, A_i_ = random effect of animal, P_j_ = fixed effect of period, T_k_ = fixed effect of treatment (PT)_jk_ = fixed effect of interaction between period and treatment, and ε_ijk_ = unexplained error. Statistical significance was set to a *p*-value < 0.05, and a tendency of difference was declared at 0.05 ≤ *p* ≤ 0.10. For abnormally distributed data, a non-parametric Wilcoxon rank-sum test and *p*-values were corrected for a false discovery rate using the Benjamini–Hochberg method, with a false discovery rate-corrected *p*-value < 0.05 being considered significant. The resulting distance matrices served as inputs for principal coordinates analysis (PCoA), and the significance of sample clustering was analyzed by permutational multivariate analysis of variance (PERMANOVA) with 9,999 permutations. The differential relative abundances of the rumen prokaryotic microbiota and its predicted metabolic categories were analyzed via linear discriminant analysis effect size (LEfSe) analysis using the Galaxy web application (51). The normalized ASV counts in each sample were used as the input for the LEfSe analysis. LEfSe uses a non-parametric factorial Kruskal–Wallis and Wilcoxon rank-sum tests followed by a linear discriminate analysis to estimate the effect size of each tax on. A significance level of *p*-value < 0.05 and an effect size threshold of 2 were applied in the trial to identify the biomarker taxa. Comparison of each exclusive network was accomplished by the use of Co-expression Differential Network Analysis (CoDiNA) (52). The built-in plugins in Gephi (v. 0.9.2) (53) were used to calculate measurements of centrality (i.e., eigenvector centrality and authority) for defining network statistics.

## Results

### Growth performance, rumen fermentation characteristics, and CH_4_ emissions

The PEO oral administration did not affect the initial BW, BW changes, or DM intake (DMI), whereas DM digestibility and final BW exhibited tendency effects (*p* = 0.081, Table 2). The average pH of the rumen fluid did not differ between the CON and PEO groups. Ammonia nitrogen concentration was lower (*p* = 0.087) in the PEO group than in the CON group. Moreover, total VFA concentration (*p* = 0.041) and molar proportion of propionate (*p* = 0.075) were significantly lower in the PEO group. The molar proportion of butyrate (*p* = 0.079) and acetate to propionate (AP) ratio (*p* = 0.086) tended to be higher in the PEO group. No interaction effect was noted between the treatment and period on growth performance and rumen fermentation characteristics, except for the final BW.

**Table 2:** Effects of *Pinus koraiensis* cone essential oil oral administration on dry matter intake, growth performance, and rumen fermentation characteristics in goats.

Item^1^	Treatment		SEM	*P-*value
	CON	PEO		
Initial BW, kg	30.0	31.2	0.92	0.220
Final BW, kg	28.9	30.2	1.00	0.211
BW change, kg	−1.10	−1.03	0.39	0.870
DM intake, kg/d	0.90	0.89	0.06	0.882
DM digestibility, %	67.0	62.7	2.32	0.081
pH	7.06	7.09	0.07	0.641
NH_3_-N, mg/dL	13.9	13.6	0.17	0.087
Total VFA, mM	33.3	28.5	1.90	0.041
**VFA, mol/100 mol**				
Acetate (A)	61.7	62.1	0.86	0.677
Propionate (P)	24.0	21.9	1.02	0.075
Butyrate	14.3	16.0	0.93	0.079
A:P ratio	2.59	2.88	0.14	0.086

SEM, standard error of the mean; CON, without PEO; PEO, *Pinus koraiensis* cone essential oil; BW, body weight; DM, dry matter; NH_3_-N, ammonia nitrogen; VFA, volatile fatty acid.

1 Over 21 days for each experiment trial.

Methane concentrations in the exhaled gas from both respiration and eructation (ppm-m, ppm/kg of DMI, and ppm/kg of DDMI) were lower in the PEO group than in the CON group; however, the difference was not statistically significant (Table 3). However, when the CH_4_ concentration was expressed as BW^0.75^, the CH_4_ concentration of the eructation was 0.79 ppm/BW^0.75^ (16.6%), which was lower (*p* = 0.079) in the PEO group than in the CON group, whereas no difference was observed in respiration. No interaction effect was noted between the treatment and period on CH_4_ emissions.

**Table 3:** Effects of *Pinus koraiensis* cone essential oil oral administration on enteric methane emission in goats.

Item^1^	Treatment		SEM	*P-*value
	CON	PEO		
**CH**_**4**_ **from respiration**				
ppm-m	21.9	21.9	1.19	0.530
ppm/BW^0.75^	1.76	1.65	0.08	0.208
ppm/kg of DMI	25.0	24.0	2.06	0.636
ppm/kg of DDMI	38.0	38.5	3.87	0.897
**CH**_**4**_ **from eructation**				
ppm-m	59.2	51.0	5.10	0.130
ppm/BW^0.75^	4.76	3.97	0.42	0.079
ppm/kg of DMI	68.3	57.9	8.65	0.252
ppm/kg of DDMI	103.9	92.7	14.6	0.466

SEM, standard error of the mean; CON, without PEO; PEO, *Pinus koraiensis* cone essential oil; CH_4_: methane; BW, body weight; DMI, dry matter intake; DDMI, digestible dry matter intake.

1 Over 21 days for each experiment trial.

### Blood metabolites

As shown in Table 4, PEO oral administration affected blood metabolite concentrations. The concentrations of albumin (*p* = 0.004), alanine transaminase/serum glutamic pyruvate transaminase (ALT/SGPT; *p* = 0.004), and creatinine (*p* = 0.011) were higher in the PEO group, whereas those of glucose (*p* = 0.065) and triglyceride (*p* = 0.032) were higher in the CON group. The concentration of aspartate aminotransferase/serum glutamic oxaloacetic transaminase (AST/SGOT), total cholesterol, inorganic phosphate, total protein, and calcium did not differ between the CON and PEO groups. No interaction effect was noted between the treatment and period on blood metabolites.

**Table 4:** Effects of *Pinus koraiensis* cone essential oil oral administration on blood metabolites concentration in goats.

Item^1^	Treatment		SEM	*P-*value
	CON	PEO		
Albumin (g/dL)	3.11	3.52	0.09	0.004
ALT/SGPT (U/L)	15.7	18.1	0.57	0.004
AST/SGOT (U/L)	78.4	78.9	6.15	0.938
Total cholesterol (mg/dL)	67.8	60.3	4.26	0.121
Creatinine (mg/dL)	0.48	0.59	0.03	0.011
Glucose (mg/dL)	83.9	76.4	3.41	0.065
Inorganic phosphate (mg/dL)	8.67	9.60	0.72	0.223
Total protein (g/dL)	6.78	6.69	0.24	0.714
Triglyceride (mg/dL)	22.8	12.4	4.36	0.032
Calcium (mg/dL)	9.51	9.50	0.24	0.959

SEM, standard error of the mean; CON, without PEO; PEO, *Pinus koraiensis* cone essential oil; ALT/SGPT, alanine transaminase/serum glutamic pyruvate transaminase; AST/SGOT, aspartate aminotransferase/serum glutamic oxaloacetic transaminase.

1 Over 21 days for each experiment trial.

### Shifts in diversity and relative abundance of rumen prokaryotic taxa

**Figure 1:**
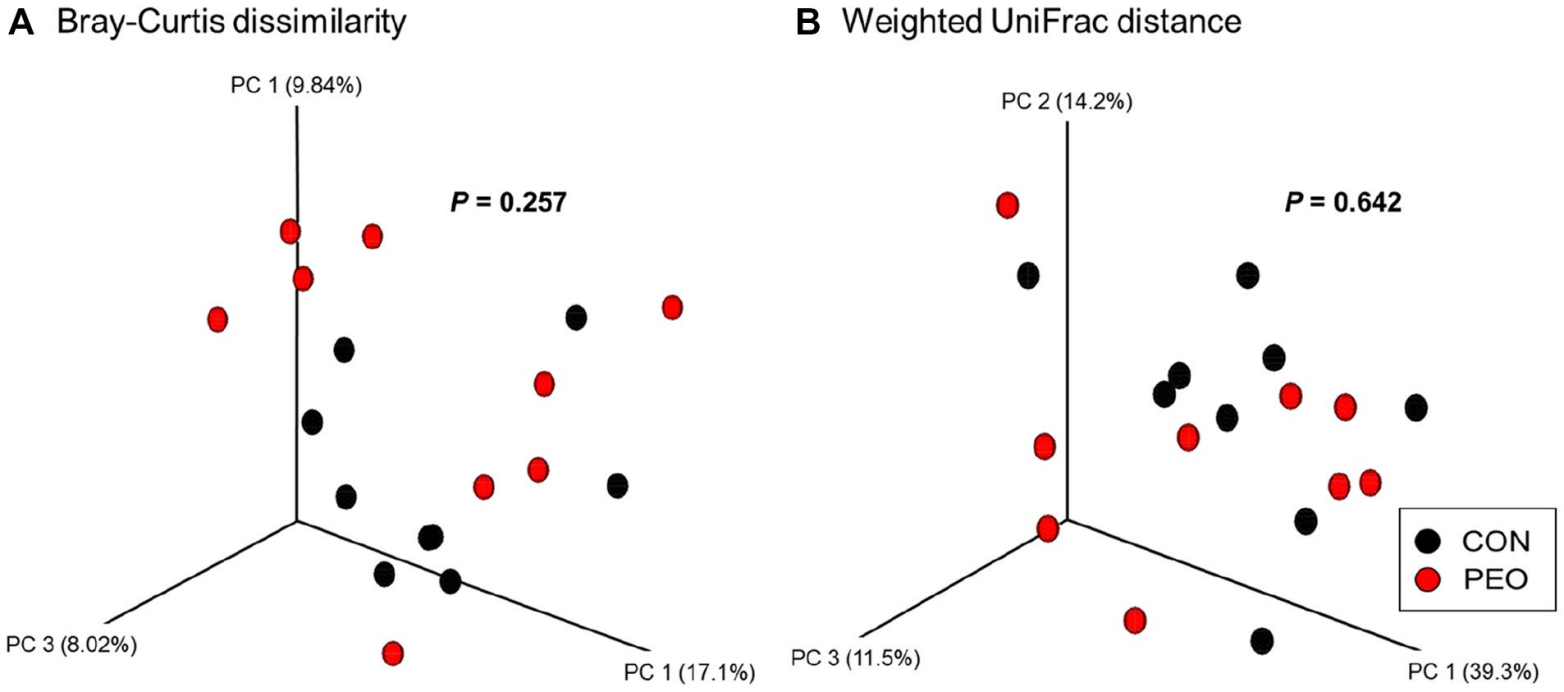
Principal coordinate analysis (PCoA) of the rumen microbiota based on the matrices of **(A)** Bray–Curtis dissimilarity and **(B)** Weighted UniFrac distance. CON, without PEO; PEO, *Pinus koraiensis* cone essential oil.

A total of 887,883 quality-controlled reads were generated (Q-score > 25), an average of 49,327 ± 5,967 (mean ± SD) sequences per sample. Good’s coverage index was 99.9% for prokaryotic sequence indicating that the sequencing depth was adequate to represent rumen microbial community. Alpha diversity measurements of the rumen prokaryotic community (Chao 1 estimates, Shannon’s index, and Simpson’s index) did not differ between the CON and PEO groups, except for Evenness, which was lower (*p* = 0.054) in the PEO group (Table 5). The PCoA and PERMANOVA revealed that compared to the CON group, PEO did not affect any of the beta diversity metrics (Bray–Curtis and Weighted UniFrac) for the rumen prokaryotic community (Figure 1).

**Table 5:** Effects of *Pinus koraiensis* cone essential oil oral administration on alpha diversity in the rumen of goats.

Item^1^	Treatment		SEM	*P-*value
	CON	PEO		
Chao1 estimate	847	804	70	0.549
Evenness	0.84	0.81	0.01	0.054
Shannon’s index	8.10	7.81	0.19	0.150
Simpson’s index	0.99	0.99	0.00	0.114

SEM, standard error of the mean; CON, without PEO; PEO, *Pinus koraiensis* cone essential oil.

1 Over 21 days for each experiment trial.

**Figure 2:**
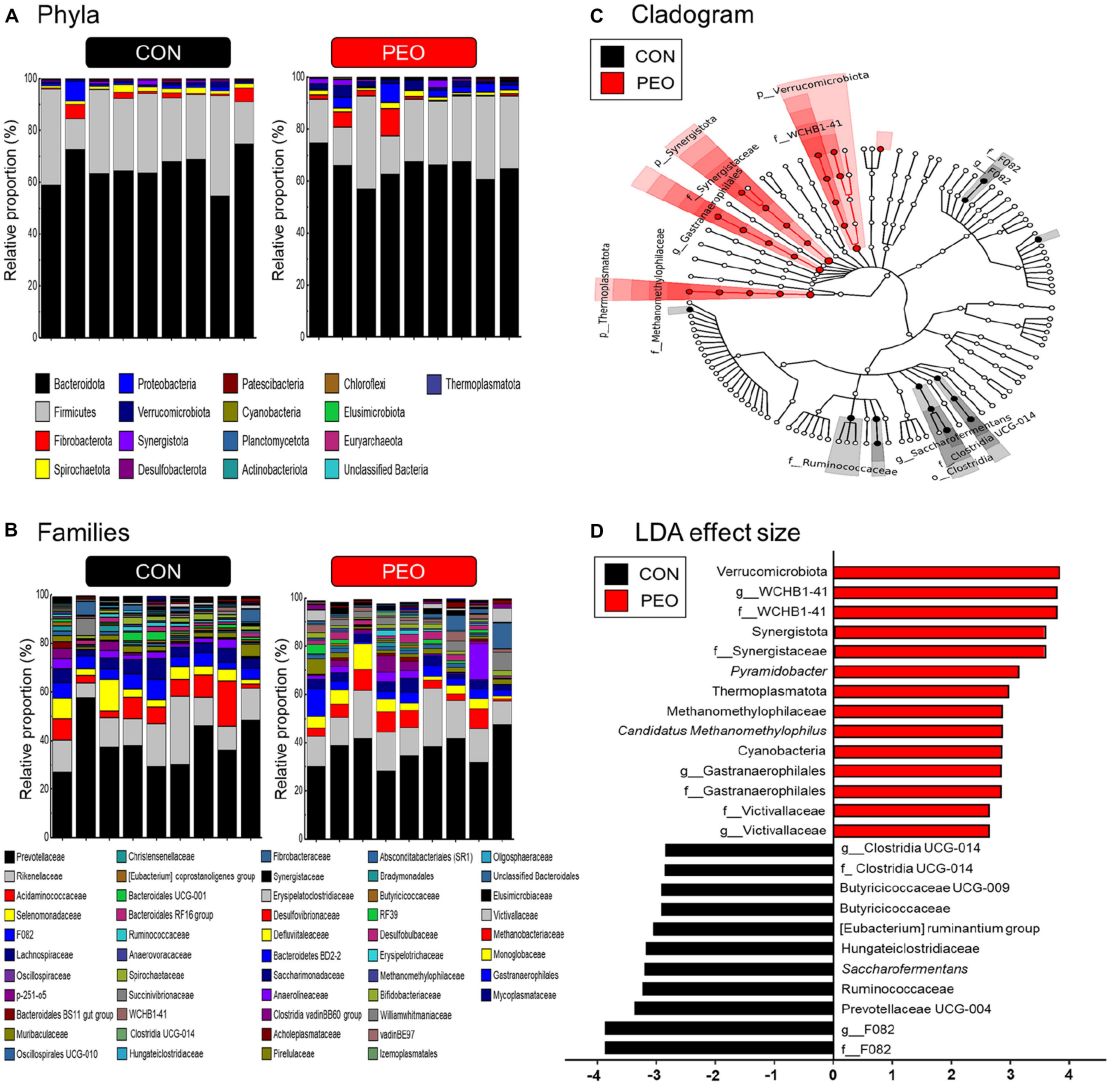
Rumen bacterial compositional profiles of goats. **(A)** Relative abundance of major bacteria phyla (relative abundance ≥ 0.01% in more than 50% animals) for all individuals. **(B)** Relative abundance of major bacteria families (relative abundance ≥ 0.01% in more than 50% animals) for all individuals. **(C)** Cladogram and **(D)** LDA effect size of significantly different taxa identified in the rumen microbiota data sets of goats based on the cutoff LDA ≥ 2.0 and *p* < 0.05. Only major classified taxa (each representing ≥ 0.01% in at least one of the dietary treatments) were visualized. CON, without PEO; PEO, *Pinus koraiensis* cone essential oil; LDA, linear discriminant analysis.

A total of 20 phyla, 109 families, and 236 genera were assigned. This study focuses on major classified taxa, which are defined as occupying over 0.01% average relative abundance in at least one of the groups. Therefore, a total of 17 phyla, 52 families, and 106 genera were identified in this study (Figures 2A,B). In total, 15 bacterial phyla were identified in the rumen samples. The most dominant phyla were the following three bacterial phyla: Bacteroidota (CON: 65.4% and PEO: 65.2%), Firmicutes (CON: 27.2% and PEO: 24.0%), and Fibrobacterota (CON: 1.95% and PEO: 2.57%; Supplementary Table S2). Two rumen archaeal phyla: Euryarchaeota (CON: 0.022% and PEO: 0.024%) and Thermoplasmatota (CON: 0.009% and PEO: 0.028%) were identified. A total of 52 bacterial families were identified, and the most dominant families were the following three bacterial families: Prevotellaceae (CON: 38.9% and PEO: 36.8%), Rikenellaceae (CON: 13.8% and PEO: 15.0%), and Acidaminococcaceae (CON: 7.60% and PEO: 5.61%). In total, 106 genera were identified, and the most dominant genera were the following three bacterial genera: *Prevotella* (CON: 31.8% and PEO: 30.9%), Rikenellaceae RC9 gut group (CON: 13.0% and PEO: 14.6%), and *Succiniclasticum* (CON: 7.46% and PEO: 5.35%; Supplementary Table S3).

The Venn diagram indicated common and exclusively identified prokaryotic taxa in the CON and PEO groups (Supplementary Figure S1). Sixteen of the 17 phyla, 47 of 52 families, and 92 of 106 genera were shared between the CON and PEO groups. At the phylum and family levels, specific taxa in the PEO group were higher than those in the CON group, whereas, at the genus level, taxa in the CON group were higher than those in the PEO group.

The LEfSe analysis identified differentially abundant microbial phyla, families, and genera between CON and PEO (Figure 2). The PEO group was differentially enriched at the phylum level than the CON group, and the enriched phyla included Cyanobacteria, Thermoplasmatota, Synergistota, and Verrucomicrobiota. At the family level, F082, Ruminococcaceae, Hungateiclostridiaceae, Butyricicoccaceae, and Clostridia UCG-014 were enriched in the CON group, whereas Victivallaceae, Gastranaerophilales, Methanomethylophilaceae, Synergistaceae, and WCHB1-41 were enriched in the PEO group. At the genus level, F082, Prevotellaceae UCG-004, *Saccharofermentans*, [Eubacterium] ruminantium group, Butyricicoccaceae UCG-009, and Clostridia UCG-014 were enriched in CON group, whereas Victivallaceae, *Gastranaerophilales*, *Candidatus Methanomethylophilus*, *Pyramidobacter*, and WCHB1-41 were enriched in PEO group.

**Figure 3:**
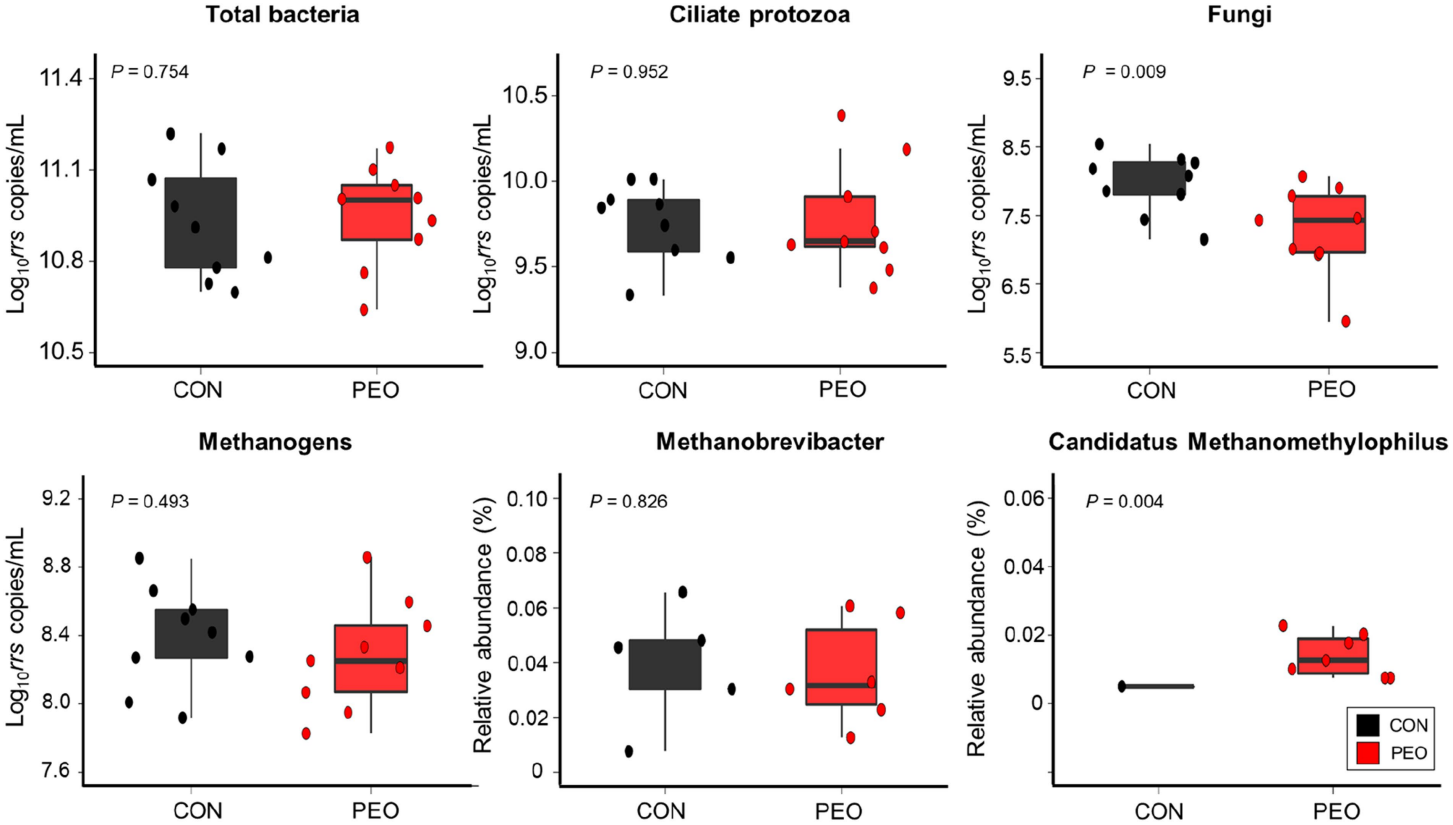
Quantification of selected bacterial species in the samples using real-time PCR and 16S rRNA analysis. The box in the box plots indicates the interquartile range (IQR; middle 50% of the data), and the middle line represents the median value. Data points represent individual animals. CON, without PEO; PEO, *Pinus koraiensis* cone essential oil.

The absolute abundances of total bacteria, ciliate protozoa, and methanogens did not show any significant differences between the CON and PEO groups, whereas the absolute abundance of fungi was significantly lower (*p* = 0.009) in the PEO group (Figure 3). No interaction effect between treatment and period on the abundance of selected bacterial groups was found.

### Prediction of rumen microbial function

**Figure 4:**
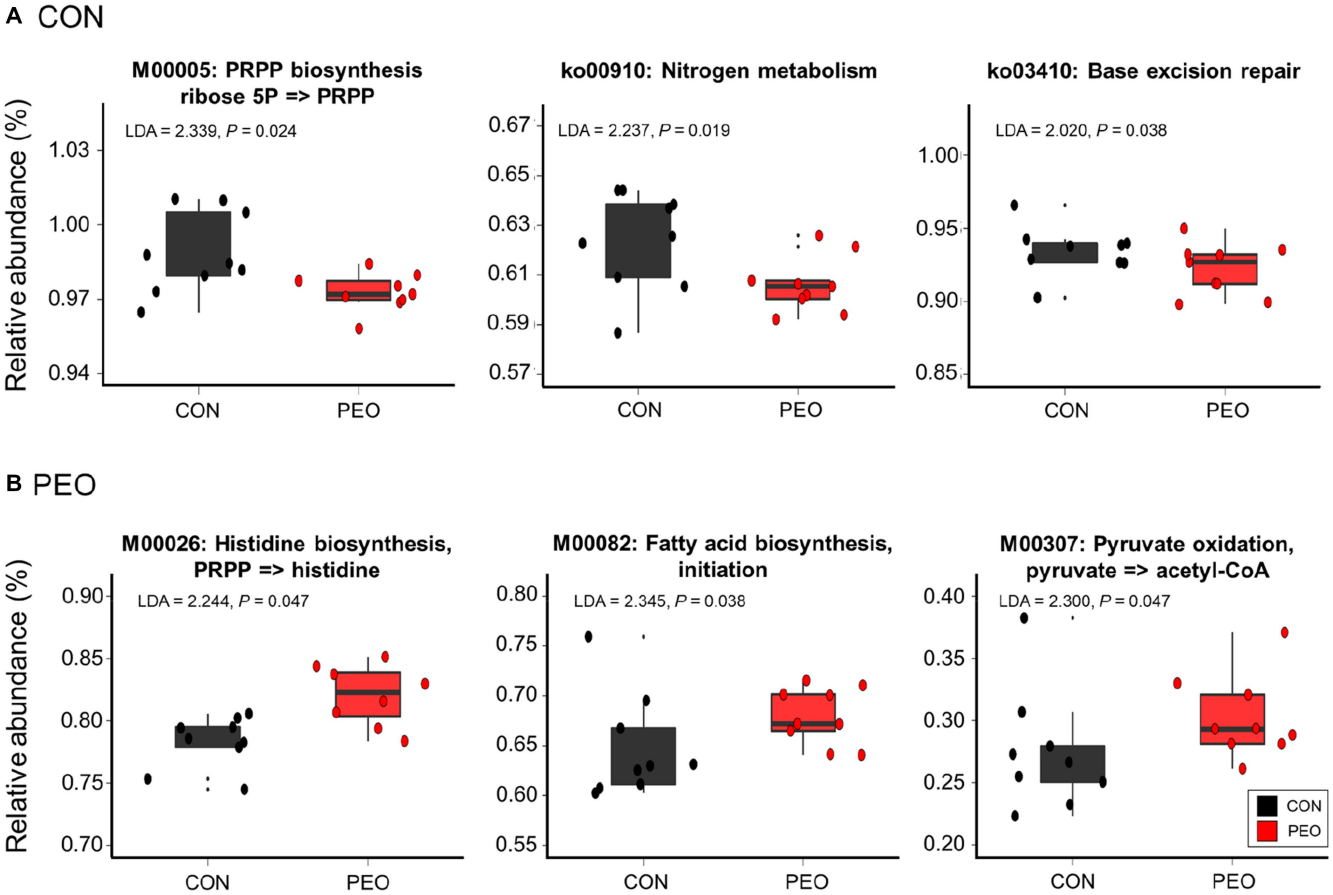
Differentially abundant KEGG modules and pathways affected by **(A)** CON and **(B)** PEO oral administration were detected using LEfSe with an LDA ≥ 2.0. Only the functional parameters accounting for ≥0.01% average relative abundance in at least one of the treatments were statistically analyzed by LEfSe. The box in the box plots indicates the interquartile range (IQR; middle 50% of the data), and the middle line represents the median value. Data points represent individual animals. CON, without PEO; PEO, *Pinus koraiensis* cone essential oil; KEGG, Kyoto Encyclopedia of Genes and Genomes; LDA, linear discriminant analysis; LEfSe, LDA effect size; PRPP, phosphoribosyl diphosphate.

No significant differences in the number of functional features predicted against eight different databases (i.e., KEGG orthologs, KEGG module, KEGG pathways, MetaCyc pathways, COG, PFAM, and EC) were found between the CON and PEO groups. The PEO group tended to have more functional features than the CON group when the KEGG orthologs database was used in the functional prediction (Supplementary Table S4); however, the overall predicted functional features were similar between the CON and PEO groups (Supplementary Figure S2). Moreover, comparative analysis using LEfSe revealed that some functions predicted based on KEGG modules and pathways were significantly enriched in the CON and PEO groups (Figure 4). In the KEGG modules, phosphoribosyl diphosphate (PRPP) biosynthesis ribose 5P => PRPP (M0005) was enriched in the CON group, whereas histidine biosynthesis, PRPP => histidine (M00026), fatty acid biosynthesis, initiation (M00082), and pyruvate oxidation, pyruvate => acetyl-CoA (M00307), were enriched in the PEO group. However, the KEGG pathways, including nitrogen metabolism (ko00910) and base excision repair (ko03410), were only enriched in the CON group.

**Figure 5:**
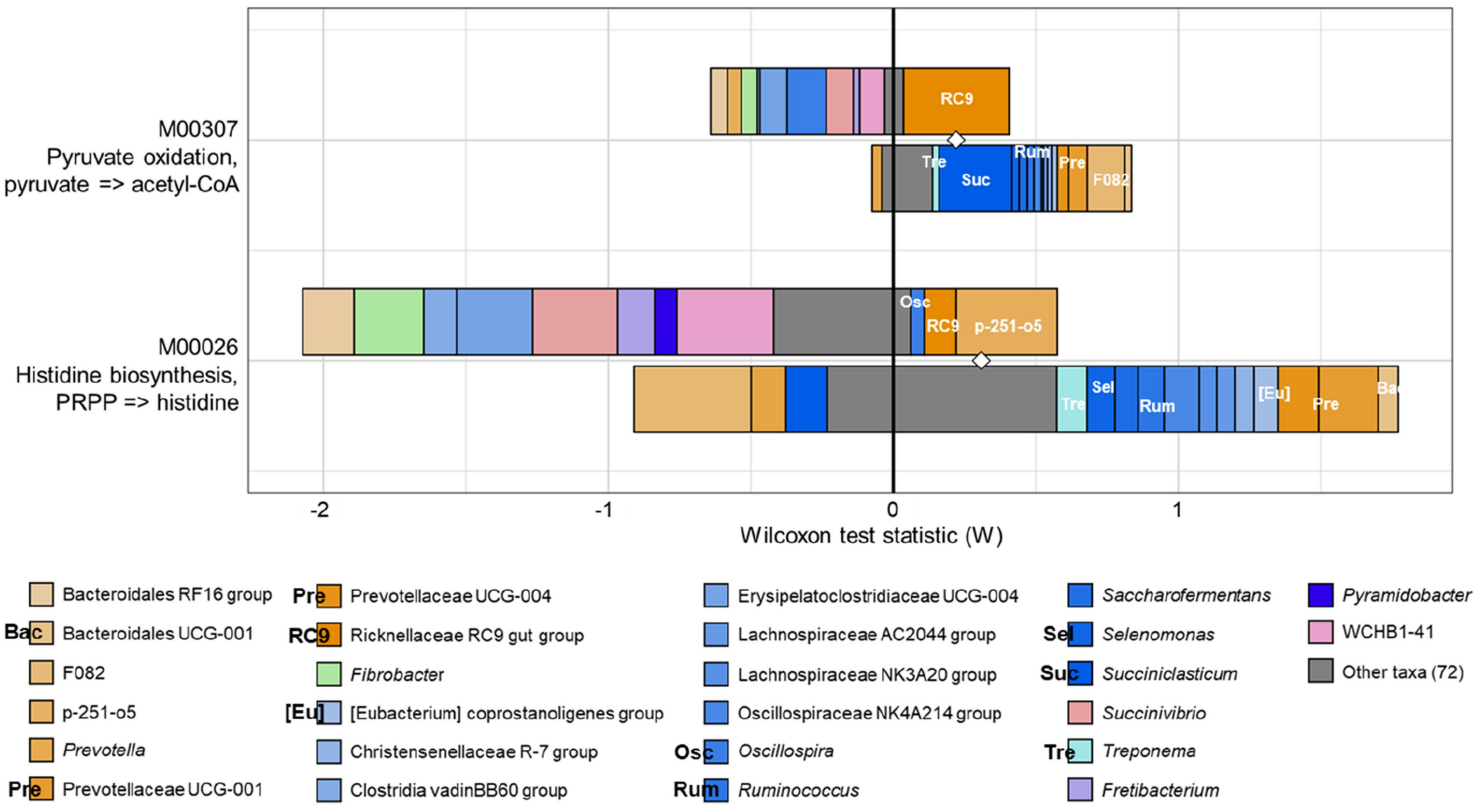
FishTaco analysis of genera contribution to KEGG modules. The x-axis represents the Wilcoxon test statistic scores, and the y-axis represents the related functions. The driving factors for each differential function transformation were divided into four parts, represented by a histogram in two directions. Rumen microbiota in the PEO group drove the increase in the corresponding functional abundance (top right). Rumen microbiota in the PEO group inhibited the proliferation in the related practical quantity (top left). Rumen microbiota in the CON group drove the increase in the related functional mass (bottom right). Rumen microbiota in the CON group inhibited proliferation in the corresponding available abundance (bottom left). Different color bars represent the related genera. The longer the bar, the greater the driving or inhibitory effect of the species on the corresponding function. Only genera accounting for ≥0.01% average relative abundance in at least one of the treatments were used. CON, without PEO; PEO, *Pinus koraiensis* cone essential oil; KEGG, Kyoto Encyclopedia of Genes and Genomes.

Having identified functional differences between the CON and PEO groups, the FishTaco analysis was applied to identify rumen prokaryotic genera estimated to drive the observed differences (Figure 5). Pyruvate oxidation and histidine biosynthesis were concurrently enriched by the Rikenellaceae RC9 gut group as the major taxonomic driver in the PEO group. In addition, *Oscillospira* and p-251-o5 specifically contributed to the enrichment of histidine biosynthesis in the PEO group. By contrast, *Treponema*, *Succiniclasticum*, Prevotellaceae UCG-001, Prevotellaceae UCG-004, and F082 in the CON group were enriched, and these genera contributed to the enrichment of pyruvate oxidation. In addition, *Treponema*, *Selenomonas*, *Ruminococcus*, Prevotellaceae UCG-001, Prevotellaceae UCG-004, and Bacteroidales UCG-001 contributed to the enrichment of histidine biosynthesis in the CON group.

### Shifts in the co-occurrence interactions of the rumen prokaryotic microbiota

**Figure 6:**
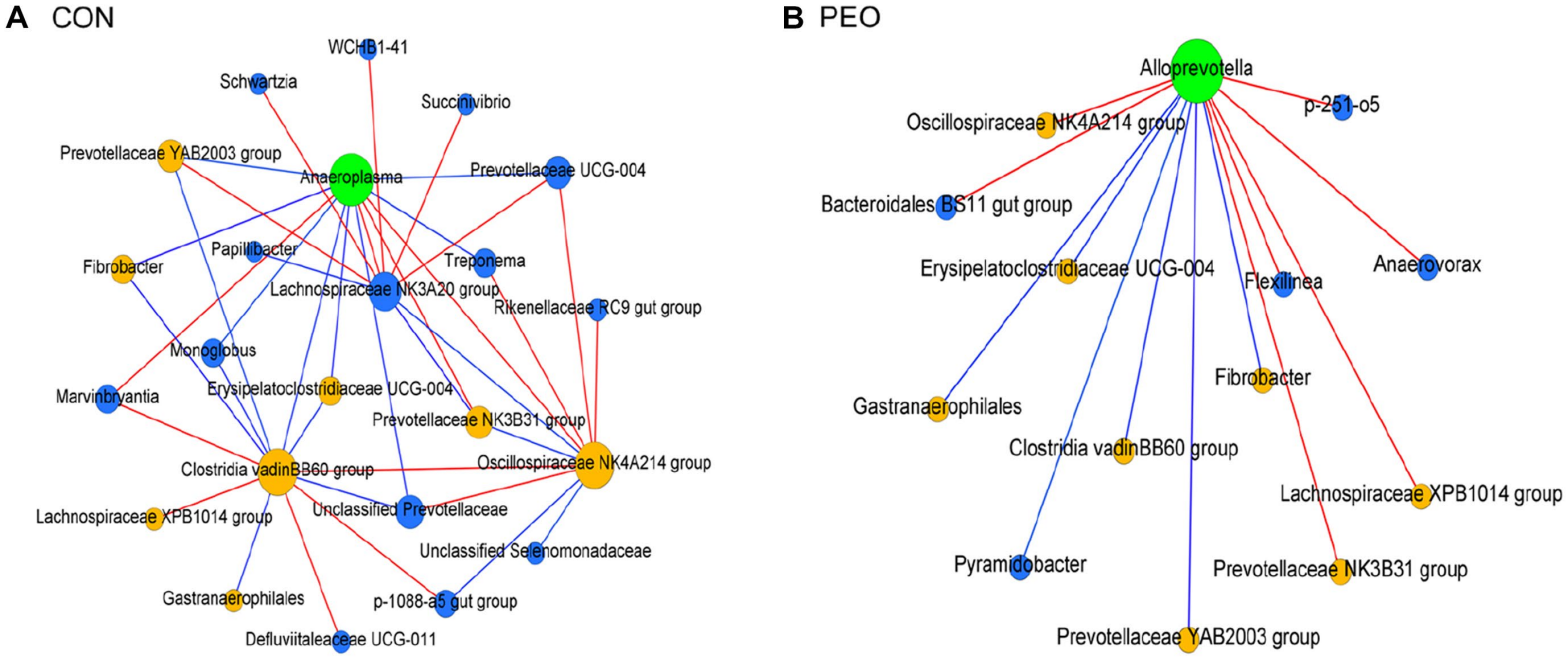
Exclusive co-occurrence and mutual exclusion microbial network in **(A)** CON and **(B)** PEO oral administration. The node color represents exclusive microbial nodes (blue) and keystone genera (green) selected based on the authority and eigenvector centrality measurements within each exclusive network. The edge color represents co-occurrence (blue) or mutual exclusive (red) interactions. Edge thickness was adjusted based on the absolute value of the correlation coefficients of each interaction. Only genera accounting for ≥0.01% average relative abundance in at least one of the treatments were used. CON, without PEO; PEO, *Pinus koraiensis* cone essential oil.

To explore the underlying relationship among rumen prokaryotic microbiota after PEO oral administration, a map of the rumen prokaryotic microbiota co-occurrence network was drawn. The co-occurrence network can be used to visualize the impact of different environmental factors on the adaptability of microbiomes. Compositional data-based network analysis revealed 23 and 14 nodes with 38 and 13 edges among the major prokaryotic genera in the CON and PEO groups (Supplementary Table S5; Figures 6A,B). A keystone genus was selected by authority and eigenvector centrality measurements within each exclusive network. In the CON group, *Anaeroplasma* was denoted as the keystone genus, which interacted with a total of 12 genera (eight co-occurrences and four mutual exclusions). In the PEO group, *Alloprevotella* was a keystone denoted as the keystone genus and interacted with a total of 13 genera (six co-occurrences and seven mutual exclusions). Moreover, the comparison of significant co-occurrence and mutual exclusion interactions between the CON and PEO groups revealed that more interactions were specifically assigned to the PEO group.

### Correlation between rumen fermentation, CH_4_ emission, and prokaryotic microbiota

**Figure 7:**
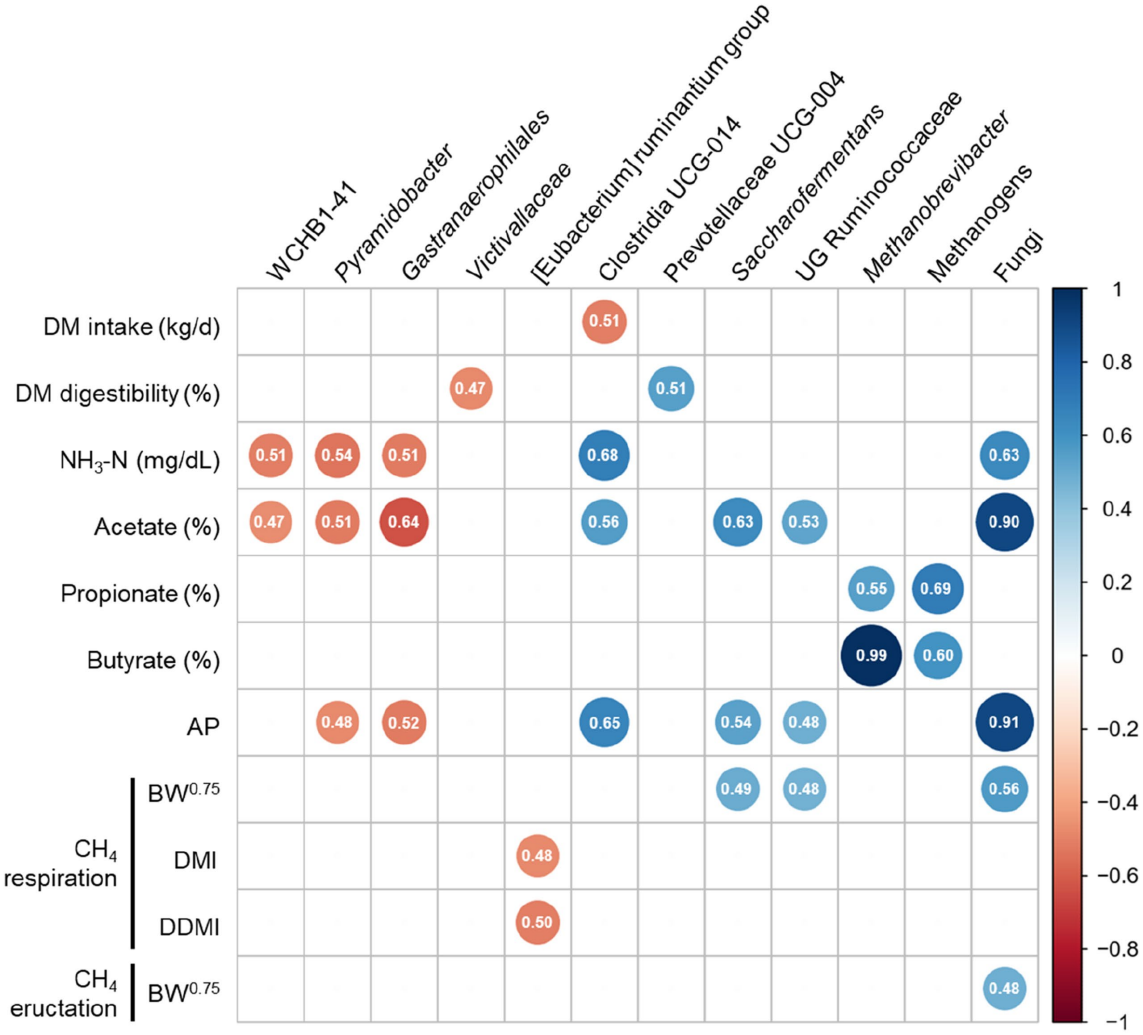
Spearman’s correlation coefficients between measurements (rumen fermentation and methane parameters) with differentially abundant prokaryotic taxa (including absolute abundance of methanogens and fungi). (|*r*| ≥ 0.5, *P* ≤ 0.05). DM, dry matter; NH_3_-N, ammonia nitrogen; AP, acetate to propionate; BW, body weight; CH_4_, methane; DMI, dry matter intake; DDMI, digestible dry matter intake.

As shown in Figure 7, a correlation analysis was performed by calculating the Spearman correlation coefficients (*r* ≥ 0.5, *p* ≤ 0.05) to identify the associations between rumen fermentation, CH_4_ emission, and prokaryotic microbiota. WCHB1-41, *Pyramidobacter*, and *Gastranaerophilales* were negatively correlated with NH_3_-N (*r* = −0.51, *p* = 0.031, *r* = −0.54, *p* = 0.021, *r* = −0.51, *p* = 0.030, respectively) and molar proportion of acetate (*r* = −0.47, *p* = 0.049, *r* = −0.51, *p* = 0.029, and *r* = −0.64, *p* = 0.004, respectively), whereas Clostridia UCG-014 and fungi were positively correlated with NH_3_-N (*r* = 0.68, *p* = 0.002 and *r* = 0.63, *p* = 0.005, respectively) and molar proportion of acetate (*r* = 0.56, *p* = 0.016 and *r* = 0.90, *p* < 0.001, respectively). Methanogens and *Methanobrevibacter* were positively correlated with the molar proportion of propionate (*r* = 0.69, *p* = 0.002 and *r* = 0.55, *p* = 0.018, respectively) and butyrate (*r* = 0.60, *p* = 0.009 and *r* = 0.99, *p* < 0.001, respectively). The genus [Eubacterium] ruminantium group was negatively correlated with CH_4_ respiration when CH_4_ concentration was expressed as DMI (*r* = −0.48, *p* = 0.046) and DDMI (*r* = −0.50, *p* = 0.033). However, fungi were positively correlated with CH_4_ eructation when CH_4_ concentration was expressed as BW^0.75^ (*r* = 0.48, *p* = 0.042).

## Discussion

In the present study, the composition of the rumen prokaryotic microbiota was altered after PEO oral administration, in accordance with the changes in the rumen environment. The lower Evenness value in the PEO group compared to the CON group indicated the presence of highly abundant genera, together with minor genera.

We observed that DM intake did not differ between the CON and PEO groups, whereas DM digestibility and rumen fermentation were lower in the PEO group than in the CON group. This effect may be due to several reasons. First, the reduction in DM digestibility and rumen fermentation may be mediated by the significantly reduced fungal abundance. Rumen fungi contribute significantly to the overall metabolism of their hosts by playing a major role in the degradation of structural polysaccharides (54), and they also have synergistic interactions with bacteria (55). Additionally, suppressed fungal abundance may provide lower feed energy to hosts, leading to a decreased total VFA concentration (56). Furthermore, rumen fungi not only produce high levels of hydrogen (H_2_), which serves as a major substrate for methanogens to ultimately produce CH_4_ (57), but they can also co-culture with methanogens and promote CH_4_ production (58). Despite a lowered CH_4_ emission in the PEO group, no significant difference in methanogen abundance was observed between the CON and PEO groups. The lack of difference in methanogen abundance could be due to the reduced abundance of fungi in the PEO group, which limited the availability of H_2_ and resulted in reduced CH_4_ production by methanogens. Alternatively, it is possible that methanogens allocated more energy toward growth and homeostasis maintenance instead of CH_4_ production due to the decreased abundance of major H_2_ producers (18). Therefore, the lowered fungi abundance after PEO oral administration might be associated with lowered DM digestibility and CH_4_ emission (CH_4_ Eructation/BW^0.75^). These results are consistent with previous *in vitro* results showing that fungi are sensitive to PEO (18). Second, *Pyramidobacter*, a member of Synergistota, was found to be more enriched in the PEO group compared to the CON group. These bacteria have been shown to enhance fiber digestion and produce acetate as the primary fermentation product (59). Furthermore, they are highly abundant in cattle that have higher CH_4_ emissions (60). However, our results were somewhat contradictory, as we found that while *Pyramidobacter* was more abundant in the PEO group, it was negatively correlated with the proportion of acetate and even lowered CH_4_ emissions. This finding is similar to that of a previous study on tucumã oil supplementation (1.0% of diet) in continuous culture, which showed that tucumã oil lowered the proportion of acetate and CH_4_ emission but increased the abundance of *Pyramidobacter* spp. (61). The exact mechanisms by which PEO increases the abundance of *Pyramidobacter* are not completely understood, but our findings suggest that a higher abundance of *Pyramidobacter* may be helpful in mitigating CH_4_ emissions.

The EO supplementation can directly reduce the abundance of ammonia-producing or proteolytic bacteria and decrease NH_3_-N concentration (62). For example, the inhibitory mechanisms of EO may result from the formation of protein complexes in the rumen, leading to a reduction in protein degradation (14). A previous *in vitro* study showed that PEO did not affect NH_3_-N concentration (18). In contrast, in the present study, NH_3_-N concentration decreased slightly in goats fed PEO. These conflicting results can be attributed to several factors. First, PEO active compounds are sparingly soluble; thus, their local concentrations may be higher, which may increase their potency *in vivo*. Furthermore, the impact of PEO compounds may be less pronounced on microbiota associated with liquid than on solid-associated species (63), as rumen content is usually filtered before inoculation *in vitro* and the main part of solids is discarded. Second, it was hypothesized that the PEO constituent could form a protein complex, as suggested by Patra and Yu (14); this complex acts as a bypass to inhibit degradation in the rumen and may allow PEO to be delivered to the lower part of the gastro-intestinal tract. This hypothesis is consistent with the results that the abundance of ciliate protozoa was not affected by PEO oral administration but reduced DM digestibility. Therefore, the decrease in NH_3_-N concentration may be associated with a reduction in the DM digestibility or ammonia-producing bacteria in the PEO group. Moreover, ammonia-producing bacteria (i.e., Butyricicoccaceae, Clostridia UCG-014, and Prevotellaceae UCG-004) and N metabolism were enriched in the CON group. Third, PEO oral administration increased the albumin concentration. Generally, total protein and albumin levels in the blood are used as indices of protein metabolism. Jahani-Azizabadi et al. (64) reported that the supplementation of a mixture of EO resulted in high albumin concentration, similar to the results of this study. However, albumin only contributes 15%–22% to export protein production (65). Therefore, the cause of PEO-induced high albumin levels is unclear. Moreover, although in the present study, we did not focus on N utilization in the present study, further research on N utilization is needed.

Essential oils have been shown to reduce CH_4_ emissions. However, the present study showed that PEO oral administration only reduced CH_4_ emissions from goats when eructation was divided into BW^0.75^, measured over 4 consecutive days. Moreover, although the CH_4_ concentration was lowered in the PEO than in the CON group, the difference was not statistically significant. The abundance of methanogens and *Methanobrevibacter* was not affected by PEO supplementation, which was consistent with previous *in vitro* results (18). Firkins and Yu (66) reported that CH_4_ reduction was not consistently accompanied by a reduction in archaeal abundance because many other factors might affect the CH_4_ production process, and the genes involved in the CH_4_ production pathway might be differentially expressed in different methanogen genera. However, in the present study, the abundance of some ruminal prokaryotic microbial genera that contribute to CH_4_ production in the rumen was affected by PEO. For example, CH_4_-producing prokaryotic taxa (Methanomethylophilaceae and *Candidatus Methanomethylophilus*) were more enriched in the PEO group than in the CON group. Interestingly, most of the *Candidatus Methanomethylophilus* were lowered in the CON group (present in only one of the nine samples), whereas they were enriched in the PEO group (present in seven of the nine samples). Recently, Martinez-Álvaro et al. (67) reported that *Candidatus Methanomethylophilus* has a different functional niche than other methanogens and is negatively correlated with CH_4_ emissions. In addition, co-abundance network analysis revealed that *Candidatus Methanomethylophilus* was clustered with the acetogens Eubacterium, *Blautia*, and Acetitomaculum, which are highly active H_2_ sinks. Another study reported that the relative abundance of *Candidatus Methanomethylophilus* was favored in cattle with low CH_4_ emissions (68). Additionally, Verrucomicrobiota, which makes a significant contribution to polysaccharide degradation (69) and is active in CH_4_ oxidation by converting CH_4_ to methanol (70), was enriched in the PEO group. These findings are consistent with the result that PEO oral administration lowered CH_4_ emissions when eructation was divided into BW^0.75^.

It was observed that PEO oral administration affected the concentration of some blood metabolites; this finding is different from the results of a previous study in dairy cows (71). Lactating dairy cows were fed a PEO mixture (PEO, garlic kernel, and brown seaweed midrib extracts at a ratio of 1:1:1) at a concentration of 0.016%, and no effect was noted on the concentrations of albumin, glucose, triglyceride, and creatine. In contrast, our study showed that PEO oral administration significantly increased the concentrations of albumin, ALT/SGPT, and creatinine. Liver health is associated with ALT/SGPT activity, and kidney health is associated with creatinine (72, 73). In the present study, higher ALT/SGPT and creatinine concentrations were observed in the PEO group, indicating that PEO oral administration affects the basic metabolic function in animals, which resulted in metabolic stress of the liver and kidneys. These results are further supported by the decreased total protein, total cholesterol, glucose, and triglyceride concentration in the PEO group. However, further studies are needed to verify the effects of PEO oral administration on blood metabolites.

In this study, exclusive keystone microbial nodes were defined using two centrality measurements. These nodes often play a unique and important role in maintaining microbial community structure and stability in the environment (74). In the PEO group, *Alloprevotella*, an acetogenic bacterium, was denoted as the keystone genus (75) and plays a key role in the regulation of lipid metabolism (76). Interestingly, *Alloprevotella* co-occurrence with *Pyramidobacter* and *Fibrobacter*, which are also acetate producers in the rumen. Furthermore, pyruvate oxidation and fatty acid biosynthesis were enriched in the PEO group. In the rumen, pyruvate can be converted by oxidation into malate, lactate, or acetyl-coenzyme A (CoA) and subsequently produce the VFAs as the energy source for the host (77). Acetyl-CoA is a precursor for the synthesis of acetate and butyrate, whereas malate and lactate are precursors of propionate synthesis (78). Therefore, the enrichment of pyruvate oxidation observed in the present study was possibly due to the upregulation of pathways involved in pyruvate metabolism and may contribute to the increase in acetate and butyrate levels in goats administered PEO. This is consistent with the results that the molar proportions of acetate and butyrate were higher in goats fed PEO. Moreover, the high molar proportions of butyrate might be associated with lowered CH_4_ emission by the relocation of electrons (79). As the electrons not consumed by CH_4_ were available for reducing NADH, this effect, in turn, mediates the conversion of acetoacetyl-CoA to butyryl-CoA and finally, to butyrate (80). In the CON group, *Anaeroplasma,* which ferments sugars primarily to formate, acetate, propionate, and succinate (81), was denoted as the keynote genus. Interestingly, *Anaeroplasma*, which is involved in starch digestion and contributes to propionate production, co-occurrence with *Prevotella*, is usually considered a multi-functional propionate producer in the rumen (82). In addition, *Selenomonas*, which produces propionate via the succinate pathways (83), and *Desulfovibrio*, which is a sulfate-reducing and VFA-utilizing genus (84), were exclusive genera in the CON group. Within the specific microbial networks altered by PEO oral administration, these prokaryotic genera may be differentially associated with other microbiota, particularly in different environmental niches, as suggested by Park et al. (85). However, since these exclusive nodes represent 65.2 to 42.9% of the overall rumen prokaryotic community, rumen fermentation and animal phenotypic differences could be derived from the shared or undefined microbial networks that occurred in each group.

The FishTaco analysis identified a potential association between the bacterial genera and metabolic pathways (50). The results showed that the Rikenellaceae RC9 gut group was a prominent taxonomic driver in pathways involving pyruvate oxidation and histidine biosynthesis in the PEO group. The Rikenellaceae RC9 gut group plays a vital role in utilizing carbohydrates to produce acetate in the rumen (86). Moreover, Wang et al. (87) and Qiu et al. (88) reported that the Rikenellaceae RC9 gut group was positively correlated with the molar proportion of acetate. Together with our findings, these results suggest that the acetyl-CoA pathway was upregulated more than the malate pathway in PEO orally administered goats. In contrast, *Treponema*, *Succiniclasticum*, *Ruminococcus*, Prevotellaceae UCG-001, Prevotellaceae UCG-004, and F082 were the taxonomic driver of pyruvate oxidation in the CON group. As reported by Rubino et al. (89), Prevotellaceae is known to play an important role in the decomposition of carbohydrates and proteins. *Ruminococcus* sp., particularly *Ruminococcus flavefacines*, normally produces succinate as a major fermentation product (90), and the *Succiniclasticum* genus ferments this succinate to produce propionate (83). The increased gene abundance by pyruvate oxidation can be explained as an increase in the growth of the taxonomic drivers in the CON group rather than that in the PEO group and is consistent with the increase in the molar proportion of propionate. Additionally, glucose concentration in the blood was high in the CON group, indicating an improvement in goat energy utilization. Propionate can be absorbed into the blood and transformed into glucose via gluconeogenesis in the livers of ruminants.

Histidine biosynthesis was enriched in the PEO group. It was hypothesized that PEO oral administration would affect histidine biosynthesis, thus producing more histidine for fermentation by rumen microbiota. When histidine was fermented by rumen microbiota, acetate and butyrate were produced, the contents of which were higher in the PEO group than in the CON group. Based on previous research, rumen bacteria (*Allisonella histaminiformans*) are known to ferment histidine and grow; however, these organisms employ glutaconyl-CoA decarboxylase and produce acetate and butyrate rather than histamine (91, 92). Moreover, *Oscillospira*, Rikenellaceae RC9 gut group, and p-251-o5 were identified as the main taxonomic driver in the PEO group. *Oscillospira* is a genus with a high likelihood of being able to secrete butyrate (93). Thus, the increase in the abundance of this genus was in accordance with the increased molar proportion of butyrate in the PEO group. However, the presence of family p-251-o5 is a “Candidatus” taxon with no current cultured representative. The relative abundances of several taxonomic drivers including *Ruminococcus* and *Treponema* were high in the CON group. A previous study reported that *Ruminococcus* is the taxa potentially contributing to enriched KEGG pathways for the amino acid biosynthesis in the rumen (94). *Treponema* can use NH_3_ in the rumen as the nitrogen source (95), which may be related to the enrichment of nitrogen metabolism in the CON group; however, further studies are needed to verify this.

In this study, it was found that oral administration of PEO reduced CH_4_ emission (eructation CH_4_/BW^0.75^) and altered blood metabolites, without affecting DM intake. However, PEO oral administration led to lower concentrations of total VFA and NH_3_-N, molar proportions of propionate, and fungal abundance in the rumen. Additionally, PEO oral administration affected the composition of some rumen prokaryotic microbiota and microbial functions. The co-occurrence and mutual exclusion of rumen prokaryotic genera were also found to be altered with PEO oral administration. Therefore, the hypothesis that PEO oral administration would alter the rumen prokaryotic community and function is mostly accepted. However, it is important to note that further research is required to gain a deeper understanding of the effects of PEO oral administration on the multi-kingdom composition and metabolism of the rumen microbiota. Advanced techniques such as multi-omics should be employed to gain a more comprehensive understanding of the potential benefits and drawbacks of PEO oral administration and to develop more effective and sustainable strategies for reducing CH_4_ emissions in livestock production. Therefore, future studies should focus on conducting multi-omics analyses, integrating rumen metabolomics and metagenomics, to unravel the underlying mechanisms of PEO and its effect on the rumen microbiome. Such studies will ultimately lead to the development of more efficient and sustainable mitigation strategies that benefit both animal health and the environment.

## Data availability statement

The datasets presented in this study can be found in online repositories. The names of the repository/repositories and accession number(s) can be found in the article/Supplementary material.

## Ethics statement

The animal study was reviewed and approved by the Gyeongsang National University Institutional Animal Care and Use Committee (protocol number: GNU-210705-E0063).

## Author contributions

YC and SJL wrote the main manuscript text, prepared tables and figures, and performed data analysis. SJL provided funding. HSK and JSE assisted statistical expertise and assisted in drafting of the manuscript. SUJ and YL analyzed and interpreted the rumen PCR results. LG and TP reviewed and edited the manuscript. JS provided rumen plasmid DNA for this study. SSL provided the necessary experimental equipment and guidance. SSL was responsible for experimental concept and design, supervised all work, and critically revised the manuscript. All authors contributed to the article and approved the submitted version.

## Funding

This study was supported by the National Institute of Animal Science, Ministry of Rural Development Administration, Republic of Korea (research project PJ01477803).

## Conflict of interest

The authors declare that the research was conducted in the absence of any commercial or financial relationships that could be construed as a potential conflict of interest.

## Publisher’s note

All claims expressed in this article are solely those of the authors and do not necessarily represent those of their affiliated organizations, or those of the publisher, the editors and the reviewers. Any product that may be evaluated in this article, or claim that may be made by its manufacturer, is not guaranteed or endorsed by the publisher.

## Supplementary material

The Supplementary material for this article can be found online at: https://www.frontiersin.org/articles/10.3389/fvets.2023.1168237/full#supplementary-material

## Abbreviations

ASVs: Amplicon sequence variants
CON: Control
CH_4_: Methane
DMD: Dry matter digestibility
KEGG: Kyoto Encyclopedia of Genes and Genomes
LEfSe: Linear discriminant analysis effect size
NH_3_-N: Ammonia nitrogen
PCA: Principal component analysis
PEO: *Pinus koraiensis* cone essential oil
PERMANOVA: Permutational multivariate analysis of variance
VFA: Volatile fatty acid
