# Supplementary material for: Oral administration of Pinus koraiensis cone essential oil reduces rumen methane emission by altering the rumen microbial composition and functions in Korean native goat (Capra hircus coreanae)
Source: Front Vet Sci. 2023 May 18;10:1168237. doi: 10.3389/fvets.2023.1168237 (PMC10234127; doi:10.3389/fvets.2023.1168237)
Supplement: Supplementary file 1 [file Data_Sheet_1.pdf]

Table S1. Primers (F = forward, R = reverse) for real-time PCR assay

Target species	Primer sequence (5′→3′)	Size (bp)[a]	Reference
Total bacteria	F: CGGCAACGAGCGCAACCC<br>R: CCATTGTAGCACGTGTGTAGCC	130	Denman and McSweeney (2006)
Ciliate protozoa	F: GCTTTCGWTGGTAGTGTATT<br>R: CTTGCCCTCYAATCGTWCT	223	Sylvester et al. (2004)
Fungi	F: GAGGAAGTAAAAGTCGTAACAAGGTTTC<br>R: CAAATTCACAAAGGGTAGGATGATT	120	Denman and McSweeney (2006)
Methanogens	F: CCGGAGATGGAACCTGAGAC<br>R: CGGTCTTGCCCAGCTCTTATTC	~160	Zhou et al. (2009)

[a]bp, base pair. F: forward; R: reverse.

Table S2. Comparison of rumen bacterial phylum[1] between CON and PEO in goats

Item	Treatments		SEM	*P*-value[2]	FDR[3]
	CON	PEO			
Bacteroidota	65.4	65.2	1.89	0.863	0.891
Firmicutes	27.2	24.0	2.74	0.387	0.730
Fibrobacterota	1.95	2.57	0.94	0.863	0.891
Spirochaetota	1.67	1.47	0.19	0.596	0.844
Proteobacteria	1.56	2.58	0.76	0.297	0.721
Verrucomicrobiota	0.757	1.992	0.29	0.007	0.100
Synergistota	0.503	1.217	0.23	0.027	0.154
Desulfobacterota	0.353	0.251	0.06	0.331	0.721
Patescibacteria	0.302	0.275	0.09	0.723	0.891
Planctomycetota	0.058	0.084	0.02	0.451	0.767
Actinobacteriota	0.055	0.029	0.02	0.752	0.891
Chloroflexi	0.048	0.045	0.02	0.322	0.721
Cyanobacteria	0.037	0.148	0.04	0.045	0.193
Elusimicrobiota	0.026	0.051	0.02	0.579	0.844
Euryarchaeota	0.022	0.024	0.01	0.891	0.891
Unclassified Bacteria	0.014	0.014	0.00	0.339	0.721
Thermoplasmatota	0.009	0.028	0.00	0.012	0.100

SEM: standard error of mean; FDR: false discovery rates; CON: without PEO; PEO: *Pinus koraiensis* cone essential oil.

[1]Relative abundance of major bacteria phylum (relative abundance ≥ 0.01% in more than 50% animals) for all individuals.

[2] *P*-value obtained from non-parametric Wilcoxon rank-sum test.

[3]False discovery rate-adjusted *P*-value.

Table S3. Comparison of rumen bacterial genera[1] between CON and PEO in goats

Item	Treatments		SEM	*P*-value[2]	FDR
	CON	PEO			
Prevotella	31.8	30.9	2.81	0.931	1.000
Rikenellaceae RC9 gut group	13.0	14.6	1.75	0.340	0.784
Succiniclasticum	7.46	5.35	1.32	0.387	0.819
F082	5.38	4.09	0.75	0.050	0.444
Prevotellaceae UCG-001	3.00	2.32	0.40	0.297	0.769
Muribaculaceae	2.17	2.07	0.48	0.489	0.849
Fibrobacter	1.95	2.57	0.94	0.863	0.995
Prevotellaceae UCG-003	1.64	1.66	0.31	0.546	0.849
Treponema	1.57	1.22	0.22	0.297	0.769
Selenomonas	1.52	1.20	0.29	0.796	0.993
Bacteroidales UCG-001	1.51	1.20	0.37	0.666	0.906
Bacteroidales RF16 group	1.48	2.26	0.25	0.050	0.444
Quinella	1.40	1.32	0.48	0.666	0.906
Oscillospiraceae NK4A214 group	1.17	0.69	0.27	0.222	0.739
p-251-o5	1.14	1.85	0.64	0.258	0.739
Lachnospiraceae ND3007 group	1.03	1.11	0.16	1.000	1.000
Unclassified_Prevotellaceae	1.03	1.00	0.17	1.000	1.000
Veillonellaceae UCG-001	1.03	0.99	0.22	1.000	1.000
UCG-010	1.01	1.16	0.19	0.605	0.876
[Eubacterium] coprostanoligenes group	0.848	0.557	0.148	0.258	0.739
Christensenellaceae R-7 group	0.806	0.525	0.219	0.387	0.819
Ruminococcus	0.806	0.513	0.126	0.050	0.444
Succinivibrio	0.766	1.727	0.545	0.222	0.739
Erysipelatoclostridiaceae UCG-004	0.711	1.544	0.415	1.000	1.000
WCHB1-41	0.655	1.782	0.259	0.004	0.141
Prevotellaceae NK3B31 group	0.588	0.453	0.163	0.796	0.993
Lachnospiraceae AC2044 group	0.583	0.299	0.119	0.136	0.655
Bacteroidales BS11 gut group	0.565	0.593	0.246	0.666	0.906
Saccharofermentans	0.562	0.282	0.075	0.031	0.444
Prevotellaceae UCG-004	0.552	0.108	0.066	0.000	0.017

Butyrivibrio	0.448	0.283	0.072	0.190	0.720
Rikenellaceae U29-B03	0.434	0.350	0.060	0.340	0.784
Anaeroplasma	0.416	0.506	0.178	0.863	0.995
Anaerovibrio	0.408	0.393	0.089	0.863	0.995
Clostridia UCG-014	0.391	0.377	0.111	0.050	0.444
Anaerovorax	0.382	0.310	0.060	0.546	0.849
Fretibacterium	0.374	0.849	0.225	0.136	0.655
Unclassified Succinivibrionaceae	0.373	0.426	0.176	0.376	0.819
Clostridia vadinBB60 group	0.370	0.696	0.142	0.340	0.784
[Eubacterium] ruminantium group	0.366	0.175	0.058	0.031	0.444
Unclassified Lachnospiraceae	0.362	0.313	0.078	0.136	0.655
Succinivibrionaceae UCG-002	0.359	0.279	0.194	0.627	0.886
[Ruminococcus] gauvreauii group	0.344	0.190	0.064	0.436	0.849
Desulfovibrio	0.318	0.194	0.056	0.297	0.769
Oscillospiraceae UCG-002	0.304	0.345	0.097	0.895	1.000
Lachnospiraceae NK3A20 group	0.283	0.129	0.068	0.136	0.655
Lachnoclostridium	0.276	0.231	0.058	0.546	0.849
Mycoplasma	0.235	0.081	0.090	0.480	0.849
Rikenellaceae SP3-e08	0.233	0.050	0.069	0.212	0.739
Papillibacter	0.219	0.288	0.068	0.480	0.849
Prevotellaceae YAB2003 group	0.188	0.360	0.158	0.190	0.720
Oscillospira	0.187	1.585	0.851	0.146	0.671
Schwartzia	0.186	0.179	0.044	0.436	0.849
Unclassified Selenomonadaceae	0.182	0.200	0.073	0.536	0.849
Absconditabacteriales (SR1)	0.176	0.185	0.064	0.436	0.849
Lachnospiraceae XPB1014 group	0.160	0.072	0.038	0.077	0.544
Bacteroidetes BD2-2	0.159	0.127	0.047	0.330	0.784
Pyramidobacter	0.129	0.367	0.053	0.012	0.250
Pseudobutyrivibrio	0.127	0.118	0.030	0.796	0.993
Candidatus Saccharimonas	0.126	0.088	0.034	0.330	0.784
Lachnospiraceae UCG-008	0.110	0.068	0.025	0.258	0.739
Moryella	0.104	0.076	0.031	0.479	0.849
Ruminococcaceae	0.103	0.017	0.027	0.069	0.521

Marvinbryantia	0.100	0.061	0.031	0.368	0.819
vadinBE97	0.094	0.152	0.039	0.258	0.739
[Eubacterium] nodatum group	0.092	0.099	0.034	1.000	1.000
Defluviitaleaceae UCG-011	0.085	0.036	0.017	0.111	0.655
Oscillospiraceae UCG-005	0.077	0.062	0.020	0.508	0.849
Alloprevotella	0.074	0.088	0.041	0.479	0.849
Sharpea	0.065	0.011	0.015	0.187	0.720
[Eubacterium] xylanophilum group	0.061	0.062	0.012	0.825	0.995
p-1088-a5 gut group	0.058	0.084	0.014	0.489	0.849
Acholeplasmataceae NED5E9	0.055	0.034	0.019	0.401	0.834
Oribacterium	0.051	0.032	0.017	0.136	0.655
Flexilinea	0.048	0.045	0.009	0.284	0.769
Unclassified Oscillospiraceae	0.040	0.048	0.024	0.860	0.995
Lachnospiraceae NK4A136 group	0.040	0.022	0.011	0.850	0.995
Candidatus Soleaferrea	0.040	0.065	0.018	0.785	0.993
Unclassified Anaerovoracaceae	0.039	0.043	0.026	0.929	1.000
Gastranaerophilales	0.037	0.148	0.014	0.047	0.444
Ruminiclostridium	0.037	0.049	0.038	0.965	1.000
Monoglobus	0.030	0.055	0.015	0.536	0.849
Roseburia	0.028	0.050	0.013	0.559	0.849
Acetitomaculum	0.027	0.034	0.014	0.721	0.968
Elusimicrobium	0.024	0.046	0.010	0.571	0.853
Methanobrevibacter	0.022	0.024	0.019	0.785	0.993
RF39	0.021	0.012	0.008	0.579	0.853
Family XIII AD3011 group	0.021	0.017	0.007	0.536	0.849
Ruminococcaceae UCG-001	0.020	0.144	0.005	0.561	0.849
V9D2013_group	0.020	0.022	0.071	1.000	1.000
Sphaerochaeta	0.020	0.021	0.010	0.965	1.000
Paraprevotella	0.018	0.013	0.007	0.965	1.000
Prevotellaceae	0.018	0.079	0.004	0.785	0.993
[Eubacterium] hallii group	0.018	0.013	0.029	1.000	1.000
Butyricicoccaceae UCG-009	0.017	0.006	0.007	0.069	0.521
[Eubacterium] brachy group	0.016	0.007	0.007	0.106	0.655

Shuttleworthia	0.015	0.027	0.004	0.963	1.000
Bradymonadales	0.014	0.023	0.004	0.258	0.739
Desulfobulbus	0.011	0.010	0.009	0.856	0.995
Prevotellaceae Ga6A1 group	0.010	0.015	0.009	0.611	0.876
Unclassified Desulfovibrionaceae	0.008	0.016	0.003	0.187	0.720
Mailhella	0.002	0.007	0.006	0.160	0.705
Victivallaceae	0.002	0.037	0.006	0.007	0.176
Candidatus_Methanomethylophilus	0.001	0.011	0.002	0.004	0.141

SEM: standard error of mean; FDR: false discovery rates; CON: without PEO; PEO: *Pinus koraiensis* cone essential oil.

[1]Relative abundance of major bacteria genera (relative abundance ≥ 0.01% in more than 50% animals) for all individuals.

[2] *P*-value obtained from non-parametric Wilcoxon rank-sum test.

[3]False discovery rate-adjusted *P*-value.

Table S4. Summary of alpha diversity measurements of the rumen microbiota for the dietary treatments

Item	Treatment		SEM	*P* value
	CON	PEO		
KEGG orthologs	4,213	4,300	138	0.550
KEGG modules	252	255	2.95	0.363
KEGG pathways	131	132	1.41	0.665
MetaCyc pathways	295	297	6.70	0.775
COG	3,536	3,626	64.2	0.202
PFAM	5,227	5,321	113	0.435
EC	1,443	1,466	33.4	0.519

SEM: standard error of the mean; amplicon sequence variant; CON: without PEO; PEO: *Pinus koraiensis* cone essential oil; KEGG: kyoto encyclopedia of genes and genomes; EC: enzyme classification; COG: clusters of orthologous genes; PFAM: protein families;

[1]Over 21 d for each experiment trial.

Table S5. Exclusive network statistics of rumen microbiota between CON and PEO.

Item	Treatments	
	CON	PEO
Nodes	23	14
Total edges	38	13
Positive	20	6
Negative	18	7
Positive (%)	52.6	46.2
Negative (%)	47.4	53.8
Abundance of exclusive node (%)	65.2	42.9
Network diameter	4	2
Graph density	0.15	0.143
Modularity	0.286	0
No. of communities	3	0
Average clustering coefficient	0.744	0
Best centrality node	*Anaeroplasma*	*Alloprevotella*

CON: without PEO; PEO: *Pinus koraiensis* cone essential oil.

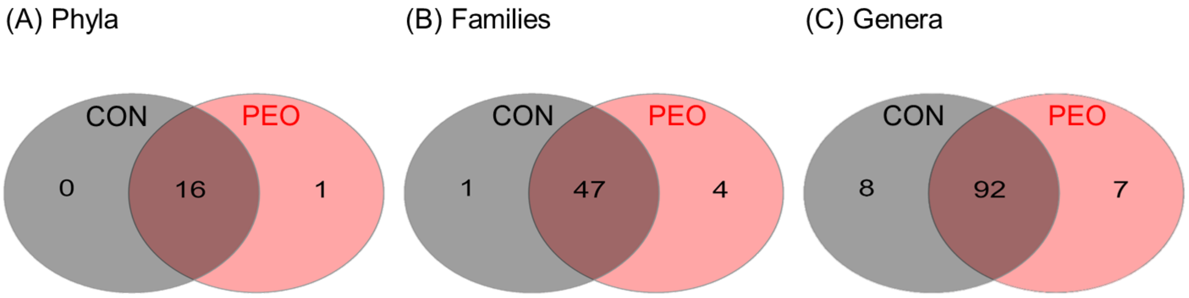

Fig. S1 The number of shared (A) phyla, (B) families, and (C) genera between the two treatments and exclusively found in each treatment were visualized using a Venn diagram.

CON: without PEO; PEO: *Pinus koraiensis* cone essential oil.

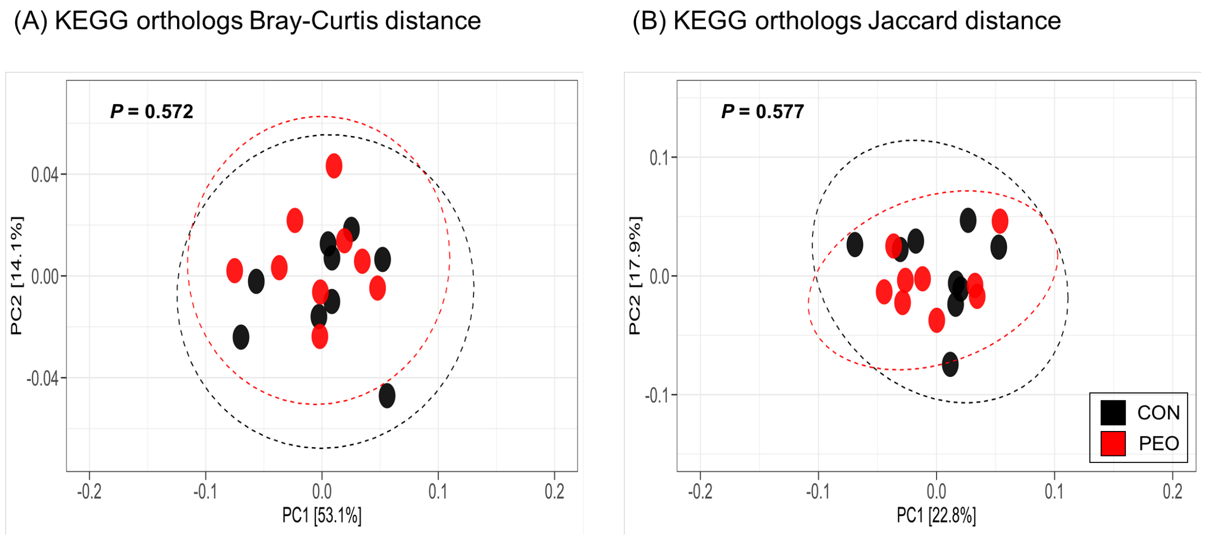

Fig. S2 Principal component analysis plots based on (A) Bray-Curtis and (B) Jaccard distance comparing the overall functional features of the rumen microbiota of prokaryotes. Functional features were predicted using PICRUSt2 database. *P*-values were based on PERMANOVA (9,999 permutations).

CON: without PEO; PEO: *Pinus koraiensis* cone essential oil; KEGG: kyoto encyclopedia of genes and genomes.
